# Topic Editorial on Photonic and Optoelectronic Devices and Systems

**DOI:** 10.3390/mi15121481

**Published:** 2024-12-09

**Authors:** Giuseppe Brunetti, Muhammad A. Butt

**Affiliations:** 1 Optoelectronics Laboratory, Department of Electrical and Information Engineering, Politecnico di Bari, Via Orabona, 4, 70125 Bari, Italy; 2 Institute of Microelectronics and Optoelectronics, Warsaw University of Technology, Koszykowa 75, 00-662 Warsaw, Poland

## 1. Introduction

Photonic and optoelectronic devices and systems represent a transformative paradigm in modern technology, exploiting the manipulation and utilization of light for diverse applications across various industries. These systems leverage the fundamental principles of photonics, incorporating the generation, detection, and modulation of photons, to achieve unparalleled speed, efficiency, and precision in comparison to traditional electronic counterparts. As the demand for high-performance systems continues to rise, photonics offers a clear advantage, particularly in fields such as communications [1], space [2], sensing [3,4], imaging [5], and quantum technologies [6].

Compared to traditional electronic devices, photonic systems offer several advantages. First, they can achieve higher data rates due to the broad bandwidth of optical signals. Second, they exhibit lower energy consumption, particularly in data transmission and storage applications, owing to the efficiency of light-based components [7]. Furthermore, the miniaturization potential of photonic devices allows for the integration of complex functionalities in compact, lightweight packages, which is crucial for applications in mobile and portable technologies.

The market for photonic devices is rapidly expanding, driven by advancements in integrated photonics, quantum computing, and LiDAR systems, all of which promise to revolutionize industries, including space, telecommunications, and autonomous vehicles, with a growth rate of 20–40% per year [8].

The benefits and market demands are driving research towards achieving ever-higher performance by developing new devices, exploiting innovative beam dynamics, engineering advanced structures, and novel system architectures. The goal is to maximize performance while maintaining a balance in size, weight, and power (SWaP). As technology evolves, there is an increasing need to push the boundaries of what is possible in terms of speed, efficiency, and functionality. This includes optimizing existing materials and processes to deliver enhanced capabilities, as well as innovating new solutions that can meet the challenges of size, weight, and power limitations, which are critical factors in the competitiveness of modern technologies.

## 2. Beam Dynamics

The beam dynamics covered in this topic explore advanced optical techniques such as the Hall effect in optical vortices [9] and diffraction-free beam formation [10], aiming at dynamically adjustable beam manipulation. These studies contribute to the understanding of optical vortex behavior and the development of advanced beam-shaping techniques.

Kotlyar et al. investigated the tight focusing of an optical vortex with an integer topological charge (TC) and linear polarization [9]. The authors demonstrated that the longitudinal components of the spin angular momentum (SAM) and orbital angular momentum (OAM) were preserved independently during beam propagation. Specifically, the SAM longitudinal component remained zero, while the OAM longitudinal component equalled the product of the beam power and the TC. This conservation of SAM and OAM led to the emergence of spin and orbital Hall effects. The spin Hall effect was manifested as the separation of regions with opposite signs of the SAM longitudinal component. The orbital Hall effect was marked by the division of areas with different rotational directions of the transverse energy flow (clockwise vs. counterclockwise). Notably, these distinct regions occurred in four localized spots near the optical axis, regardless of the TC value.

The authors also showed that the total energy flux passing through the focal plane was less than the total beam power. This discrepancy arose because part of the energy propagated along the focus surface, while the remainder crossed the focal plane in the opposite direction. Furthermore, it was found that the longitudinal component of the total angular momentum (AM) vector did not equal the sum of SAM and OAM. Interestingly, there was no SAM term in the expression for the angular momentum density, implying that SAM and OAM are independent quantities. The distributions of the longitudinal components of AM and SAM were crucial in characterizing the spin and orbital Hall effects at the focal point, respectively.

Khorin et al. proposed an approach to create a diffraction-free beam with a complex structure by iteratively calculating a set of primitives for the ring spatial spectrum [10]. In addition, the complex transmission function of diffractive optical elements (DOEs) was optimized to generate basic diffraction-free patterns, such as squares and triangles. By superimposing these optimized DOEs, along with deflecting phases (forming a multi-order optical element), a diffraction-free beam with a more intricate transverse intensity distribution was produced that reflects the combination of these primitives.

This method offered two key advantages. First, it allowed for rapid convergence to an acceptable error in the calculation of optical elements that form simple distributions, as opposed to more complex ones, especially during the initial iterations. Second, it enabled easy reconfiguration. Since the complex distribution was composed of individual primitive components, it can be swiftly or dynamically adjusted using a spatial light modulator (SLM) to manipulate and rotate these components. Numerical simulations of this approach were experimentally validated, confirming its effectiveness.

## 3. Sensing Technologies

This topic explores innovations in optical sensing technologies, with a particular focus on waveguide optical ring resonators, demonstrating their versatility in both technology platforms (dielectric and plasmonic) and target applications.

Kazanskiy et al. examined ring resonator structures built on three prominent platforms: silicon-on-insulator (SOI), polymers, and plasmonics [11]. These platforms offer remarkable versatility, enabling compatibility with various fabrication techniques and seamless integration with other photonic components. This adaptability provides significant flexibility in the design and implementation of diverse photonic devices and systems. Optical ring resonators are typically small and compact, making them ideal for incorporation into dense photonic circuits. Their small size not only allows for high device density but also facilitates integration with other optical components, paving the way for the development of complex and multifunctional photonic systems. Among these platforms, plasmonic ring resonators stand out due to their exceptional sensitivity and tiny footprint. However, the primary challenge lies in the intricate fabrication requirements of these nanoscale devices, which currently hinder their widespread commercialization.

Zheng et al. investigated a novel semi-buried optical waveguide ring resonator (SOWRR) structure for next-generation membrane-free acoustic sensors [12]. This design leveraged air as the upper cladding medium, allowing the evanescent field to interact with acoustic waves and induce measurable changes in the resonator’s frequency. The authors established an acoustic sensing model and optimized the SOWRR parameters to maximize the Q factor (8.33 × 10^6^) and resonance depth. Simulations demonstrated a wide frequency response range (1 Hz to 1.58 MHz) and a minimum detectable sound pressure of 7.48 μPa using a laser with a linewidth of 1 kHz.

Furthermore, Butt presented a numerical analysis of a plasmonic sensor based on a metal-insulator-metal (MIM) waveguide, designed for the detection of tuberculosis (TB)-infected blood plasma [13]. Directly coupling light to the nanoscale MIM waveguide is challenging; therefore, the author suggested two Si_3_N_4_ mode converters integrated into the sensor to facilitate efficient coupling. These converters enabled the conversion of the dielectric mode into a plasmonic mode, which then propagates through the MIM waveguide via the input mode converter. At the output port, the plasmonic mode was converted back to the dielectric mode using the output mode converter. The proposed sensor was specifically tailored to detect TB-infected blood plasma, which has a slightly lower refractive index compared to normal blood plasma. To effectively distinguish between the two, a high-sensitivity sensing device was crucial. The proposed device achieved a sensitivity of approximately 900 nm/RIU and a figure of merit of 11.84, demonstrating its potential for precise detection of TB-infected blood plasma.

## 4. Photonic Devices and Systems

This topic highlights recent advancements in photonic devices and related systems for a wide range of applications, such as telecommunications [14,15], computing [16], and quantum [17].

Rehman et al. introduced an innovative optical switch design based on guided-mode resonance (GMR) within a 3D photonic-crystal structure [14]. The optical switching mechanism was explored in a dielectric slab-waveguide configuration, operating in the near-infrared spectrum at a telecom wavelength of 1.55 µm. The switch functions by utilizing the interference between two signals: the data signal and the control signal. The data signal was coupled into the optical structure and selectively filtered through guided-mode resonance, while the control signal is index-guided within the structure. The amplification or attenuation of the data signal was controlled by adjusting the spectral properties of the optical sources and the structural parameters of the device. Initially, the parameters were optimized using a single-cell model with periodic boundary conditions, followed by a more detailed finite 3D-FDTD (Finite Difference Time Domain) model of the device. The numerical simulations were carried out on an open-source FDTD platform. The results showed an optical amplification of 13.75% in the data signal, accompanied by a significant reduction in linewidth to 0.0079 µm, achieving an impressive quality factor of 114.58. The proposed optical switch demonstrated significant potential for applications in photonic integrated circuits, biomedical technologies, and programmable photonics, offering enhanced performance and tunability for future photonic systems.

Kotb et al. explored the implementation of fundamental Boolean logic operations using compact Y-shaped silicon-on-silica optical waveguides designed for operation at the telecommunications wavelength of 1.55 μm [16]. The proposed waveguide consisted of four identical slots and a microring resonator. By leveraging constructive and destructive interferences caused by phase differences in input optical beams, the authors demonstrated the realization of XOR, AND, OR, NOT, NOR, NAND, and XNOR logic functions. FDTD simulations confirmed the high performance of these operations, achieving contrast ratios (CR) in the range of 25.17–34.10 dB and operational speeds of up to 120 Gb/s.

Chen et al. explored coherent optical propagation in a photonic molecule system composed of two whispering-gallery microcavities, one of which is a spinning optomechanical cavity and the other is an auxiliary optical cavity [17]. The spinning of the optomechanical cavity induced different Sagnac effects, which influenced the evolution of optomechanically induced transparency (OMIT) and related propagation effects, such as slow and fast light phenomena. Numerical simulations revealed that the direction of spin (clockwise or counterclockwise) and the coupling between the two cavities significantly affect the slow and fast light effects, as well as the transition between them. When the spinning optomechanical cavity rotated in the clockwise direction, the slow light effect was enhanced, while counterclockwise rotation led to a more pronounced fast light effect. These results indicated that both the spinning direction and the cavity–cavity coupling parameters—along with the Sagnac frequency shifts—determined the coherent optical propagation properties.

Yoo et al. presented a radar transceiver system that incorporated photonic elements to achieve wideband capabilities for high-resolution Inverse Synthetic Aperture Radar (ISAR) imaging [15]. The design of the radar system was based on budget analysis, and field tests were conducted to assess its performance, particularly focusing on the impact of bandwidth on image resolution. The proposed radar transceiver integrated several photonic elements, including a laser diode, a dual-parallel Mach–Zehnder modulator, a phase modulator, and other conventional radar components like antennas, amplifiers, and analog-to-digital converters. These photonic components enabled the system to generate a wideband signal necessary for high-resolution ISAR imaging. Field test results demonstrated the radar’s capability to detect a small drone target at a distance of 2.5 km, with ISAR images acquired from varying bandwidths (500 MHz to 2 GHz). As expected, increasing the bandwidth improved the image resolution, confirming the effectiveness of the system’s wideband characteristics.
